# Symptom network analysis during radiotherapy in gastrointestinal cancers: A longitudinal study primarily involving esophageal cancer patients

**DOI:** 10.1016/j.apjon.2025.100786

**Published:** 2025-09-17

**Authors:** Mengjia Liu, Huiwen Ma, Yaxin Chang, Hong Li, Ruiqi Chen, Weizheng Cui, Zhaoxia Yang, Ke Wang

**Affiliations:** a Department of Nursing, The Second Affiliated Hospital of Shandong First Medical University, Tai'an, China; b School of Nursing, Shandong First Medical University, Tai'an, China; c Department of Radiation Oncology, The Second Affiliated Hospital of Shandong First Medical University, Tai'an, China

**Keywords:** Gastrointestinal cancer, Network analysis, Core symptom clusters, Quality of life, Longitudinal study

## Abstract

**Objective:**

To investigate longitudinally the core symptom clusters and their association with quality of life in patients undergoing radiotherapy for gastrointestinal cancers, with the aim of reducing symptom burden and enhancing quality of life.

**Methods:**

A longitudinal study was conducted among 130 conveniently selected patients receiving radiotherapy for gastrointestinal cancers. Symptom occurrence and quality of life were assessed using a general information questionnaire, the M.D. Anderson Symptom Inventory-Gastrointestinal Cancer Module, and the Quality of Life Scale at three time points: 7 days before radiotherapy (T0), the 15th radiotherapy session (T1), and 30 days after completion of radiotherapy (T2).

**Results:**

Network analysis revealed that loss of appetite exhibited the highest centrality strength at T0 (*r*_S_ ​= ​1.293) and T2 (*r*_S_ ​= ​1.180), and the highest betweenness at T0 (*r*_B_ ​= ​50), while fatigue demonstrated the highest strength at T1 (*r*_S_ ​= ​1.215). Statistically significant differences were observed in quality of life scores across all dimensions—excluding shortness of breath and constipation—at different time points (*P* ​< ​0.05). Core symptom cluster severity scores were negatively associated with quality of life scores at all time points (*P* ​< ​0.01).

**Conclusions:**

The emotion-energy deficiency cluster was identified as the core symptom cluster, with its severity negatively associated with quality of life. Continuous interventions targeting this cluster are essential to enhance the precision and effectiveness of symptom management and thereby improve patients’ quality of life.

## Introduction

Research shows that, in 2022, approximately 5.04 million new cases of gastrointestinal (GI) cancers were reported globally, accounting for 25.2% of all cancer cases, and approximately 3.4 million deaths, which account for 34.9% of all cancer-related deaths.^1^^,^^2^ In China, over 1 million new cases of GI cancers are diagnosed annually, accounting for approximately 40% of the global total.^3^ The mortality rate ranks highest among all malignant tumors in China, and the disease burden is significantly higher than the global average, posing a serious threat to the life and health of Chinese residents.^4^ Radiotherapy is a primary treatment for patients with GI cancers. As the disease progresses and toxic side effects accumulate, patients often experience a range of symptoms, such as pain and fatigue, which tend to appear concurrently and interact synergistically, forming symptom clusters (SCs),^5^, ^6^, ^7^ which significantly impact patients’ quality of life.^8^

In recent years, with the in-depth study of symptom clusters, scholars both domestically and internationally have extended this concept to the field of gastrointestinal tumors,^9^, ^10^, ^11^ offering new perspectives for symptom management and intervention. Su et al.^12^ demonstrated that symptom clusters of eating difficulties, digestive symptoms, and energy deficiency-psychological symptoms remained stable during radiotherapy in patients with gastrointestinal tumors. In the middle and late stages of radiotherapy, patients developed symptom clusters related to radiotherapy-induced adverse reactions. In a study by Rha et al.,^13^ cancer patients were studied during the first two cycles of adjuvant chemotherapy, where biopsychological, fatigue-cognitive, genitourinary, and gastrointestinal symptom clusters were observed during the first cycle. In the second cycle, the biopsychological and fatigue-cognitive symptom clusters merged into a single cluster. The above studies demonstrate that symptom clusters change dynamically at different stages of a patient's treatment, which prompted an investigation into how symptoms are related to each other and how these relationships evolve over time.

It has been found that GI cancer patients experience core symptoms that have a substantial impact on their physical and psychological well-being,^14^ and that targeted interventions can effectively alleviate related symptoms and enhance the efficiency of symptom management.^15^^,^^16^ Network analysis,^17^ a novel research paradigm that assumes symptoms (nodes) interact rather than exist independently, enables the quantitative assessment and visualization of associations among symptoms. Symptom network analysis offers distinct advantages over traditional cluster analysis, as it not only visually represents symptom interrelationships but also helps identify core and bridge symptoms, thereby facilitating more precise symptom management.^16^^,^^18^ Currently, network analyses in oncology primarily focus on lung,^19^^,^^20^ breast,^21^^,^^22^ and gynecological cancers,^23^ however, these studies are predominantly cross-sectional, relying on symptom assessments at a single time point, thereby limiting insights into the temporal evolution of core symptoms. In addition, most existing studies focus on chemotherapy patients, while symptom networks during radiotherapy in GI cancer patients remain underexplored. Furthermore, longitudinal identification of core symptom clusters and their association with quality of life during radiotherapy is still lacking.

Based on the existing literature and clinical observations, we hypothesize that a core symptom cluster exists among patients with gastrointestinal cancers undergoing radiotherapy, and that the severity of this cluster is negatively associated with quality of life across different treatment stages.

Therefore, the aim of this study was (1) to identify core symptom clusters during radiotherapy in patients with GI cancers using network analysis, and (2) to explore longitudinally the association between the core symptom cluster and quality of life at three time points: before radiotherapy (T0), at the 15th radiotherapy session (T1), and 30 days after completion of radiotherapy (T2), in order to provide a basis for developing targeted and efficient symptom management strategies and enhancing patients’ quality of life.

## Methods

### Design and subjects

A convenience sampling method was employed to recruit patients with GI cancers who underwent radiotherapy at the radiotherapy center of a tertiary hospital in Shandong Province between September 2023 and May 2024. The inclusion criteria were (1) confirmed diagnosis of GI cancer through pathological and histological examinations; (2) patients who completed a prescribed course of curative or definitive radiotherapy with intensity-modulated radiation therapy (IMRT); (3) aged ≥ 18 years; (4) conscious, with normal communication ability, and providing informed consent to participate. The exclusion criteria were (1) presence of other systemic malignant tumors; (2) diagnosis of psychiatric disorders; (3) coexisting vital organ dysfunction; (4) patients who received concurrent chemoradiotherapy.

### Sample size

Based on the ranked incidence of symptoms, the top 13 symptoms were included in the network analysis model. Therefore, the estimated threshold parameter of the network analysis model was 13, and the total correlation parameter^24^ was estimated according to the formula *n* ​+ ​*n* (*n* - 1) / 2. The minimum sample size was calculated to be 91. Considering a 20% attrition rate, the final sample size was determined as 114 cases.

### Survey instruments

#### General information questionnaire

Socio-demographic variables included gender, age, education level, marital status, occupation, place of residence, per capita monthly household income, method of medical expense payment, religious affiliation, smoking history, and history of alcohol. Disease-related data included body mass index (BMI), cancer diagnosis, tumor stage, total number of radiotherapy sessions, radiation dose, and whether the patient underwent chemotherapy, surgery, or developed metastases.

#### M.D. Anderson Symptom Inventory–gastrointestinal cancer (MDASI–GI)

The MDASI-GI was originally developed by the University of Texas MD Anderson Cancer Center and was culturally adapted and validated by Wang et al.^25^ for Chinese gastrointestinal cancer patients. This scale is used to assess the severity of symptoms experienced over the past 24 hours. It comprises 13 core items from the M.D. Anderson Symptom Inventory and five GI-specific symptoms: constipation, bloating, diarrhea, dysphagia, and reduced taste. Each item is rated on a 0–10 numeric scale, where 0 indicates “no symptoms or interference” and 10 represents “the worst imaginable severity or complete interference.” Higher scores indicate greater symptom burden. The Cronbach's α coefficient in this study was 0.960, indicating good reliability and validity.

#### European organization for research and treatment of cancer–quality of life core questionnaire–30 (EORTC QLQ–C30)

The EORTC QLQ–C30 is a core quality of life assessment instrument developed by the EORTC.^26^ A validated Chinese version of the QLQ–C30 was introduced in 1995 and has since been widely used among Chinese cancer populations.^27^^,^^28^ It consists of 30 items covering five functional dimensions (somatic, role, cognitive, emotional, and social functioning), three symptomatic dimensions (fatigue, pain, and nausea and vomiting), an overall quality of life dimension, and six single-symptomatic items (sleep disturbances, respiratory difficulties, loss of appetite, diarrhea, constipation, and perception of financial difficulties). Items 1–28 were scored on a 4-point Likert scale (1 ​= ​not at all; 4 ​= ​very much), while items 29 and 30 use a 7-point scale (1 ​= ​very poor; 7 ​= ​very good). Higher scores on the functioning and overall quality of life dimensions indicate better functional status and quality of life, while higher symptom scores indicate greater symptom burden and worse quality of life. The Cronbach's α coefficient in this study was 0.926, demonstrating good reliability and validity.

### Data collection

Data were collected at three time points: within 7 days before radiotherapy (T0), at the 15th radiotherapy session (T1), and 30 days after the end of radiotherapy (T2). Face-to-face surveys were conducted on site. Trained research staff explained the study purpose and procedures in detail, and written informed consent was obtained before questionnaire administration. Participants were asked to complete the questionnaires independently (self-administered), and the forms were collected immediately after completion. For patients with visual impairment, writing difficulties, or literacy barriers, the questionnaires were administered by staff who read each item aloud and recorded answers strictly according to the patient's verbal response, without interpretation or guidance. All completed questionnaires were cross-checked and double-entered into the database by two independent researchers to ensure data integrity and accuracy.

### Statistical analysis

IBM SPSS Statistics (version 26.0) and R software (version 4.0.4)^29^ were used for statistical analysis. A two-sided significance level of *α* ​= ​0.05 was adopted, and differences were considered statistically significant at *P* ​< ​0.05.

Categorical variables were statistically described using frequencies and percentages. Normally distributed continuous variables were expressed as mean ​± ​standard deviation (M ​± ​SD), whereas non-normally distributed variables were reported as median and interquartile range (P25, P75).

The “qgraph” package in R software (version 4.0.4) was utilized to construct symptom network graphs for three time nodes. In the network, symptoms were represented as nodes, and the edges between nodes represented associations; the thicker the edges, the stronger the correlation between symptoms.^30^ Centrality indicators include: (1) Strength: defined as the sum of the absolute values of the edge weights, which is used to measure the importance of the nodes in the network, and the larger its value, the greater the influence of the symptom on other symptoms, and it is the core symptom of the symptom network;^31^ (2) Closeness: is the sum of the reciprocal of the shortest path between a node and other nodes, the larger its value, the closer the symptom is to other symptoms and may be located in the center of the network; (3) Betweenness: is the number of times a node is located in the shortest paths of any two nodes, the larger the value, the more likely that the symptom is a bridging symptom.The stability of the centrality metrics was assessed by the Centrality Stability Coefficient (CS-coefficient), which indicates that the correlation between the original centrality metrics and the centrality metrics of the subset-based network is at least 0.7 when the maximum proportion is removed. A coefficient of at least 0.25 is considered acceptable, and a value greater than 0.5 is preferred, indicating good stability.^30^^,^^32^ The accuracy of edge weights was assessed using the “bootnet” package based on the bootstrap algorithm to calculate the 95% confidence interval (CI). Edge weight and centrality discrepancy test were used to further validate the stability of the symptom network at each time node.

Repeated measures ANOVA was used to assess differences in quality of life across time points. Spearman correlation analysis was used to examine the association between core symptom clusters and quality of life.

## Results

### Demographic and clinical characteristics

**Fig. 1:**
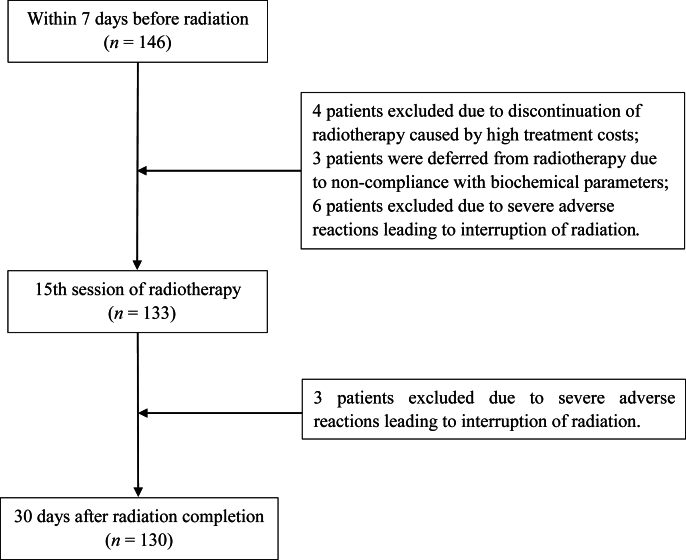
Participant flow diagram across radiotherapy time points.

A total of 146 patients were initially enrolled in this study. After exclusions due to treatment cost, biochemical non-compliance, and severe adverse reactions, 130 patients completed all three assessment points and were included in the final analysis, yielding a valid response rate of 89.0%. The detailed participant flow is illustrated in Fig. 1.

Among the 130 patients who received radiotherapy for GI cancer, 103 were male and 27 were female. The age ranged from 47 to 83 (65.02 ​± ​7.76) years old. The mean BMI was 22.51 ​± ​3.15 kg/m^2^, and the majority of tumors were classified as stage III or IV. Additional demographic and clinical details are presented in Table 1.

**Table 1:** General data of patients with GI cancer (*N* ​= ​130).

Characteristics	Group	Mean (SD)	*n*	%
Sex	Male		103	79.2
	Female		27	20.8
Age (years)	40–60	65.02 (7.76)	36	27.7
	≥ 60		94	72.3
Education level	Primary and below		51	39.2
	Junior high school		67	51.5
	High school and above		12	9.2
Marital status	Married		128	98.5
	Widowed spouse		2	1.5
Occupation	Farmer		76	58.5
	Workers		24	18.5
	Staff member		4	3.1
	Retiree		18	13.8
	Others		8	6.1
Current address	Rural		88	67.7
	Urban		42	32.3
Monthly per capita household income (yuan)	< 1000		32	24.6
	1000–2999		63	48.5
	3000–4999		28	21.5
	≥ 5000		7	5.4
Methods of payment	Medical insurance for residents		101	77.7
	Employee health insurance		29	22.3
Religious belief^a^	Yes		5	3.8
	No		125	96.2
History of smoking	Yes		58	44.6
	No		72	55.4
History of alcohol	Yes		55	42.3
	No		75	57.7
BMI (kg/m^2^)	BMI < 18.5	22.51 (3.15)	8	6.2
	18.5 ≤ BMI < 24.0		92	70.8
	24.0 ≤ BMI < 28.0		33	25.4
	BMI ≥ 28.0		3	2.3
Disease diagnosis	Esophageal cancer		98	75.4
	Colorectal cancer		26	20
	Gastric cancer		6	4.6
Tumor staging	I		1	0.8
	II		18	13.8
	III		55	42.3
	IV		56	43.1
Total number of radiotherapy		28.72 (3.35)		
Radiation dose (Gy)		56.84 (6.41)		
Chemotherapy history	Yes		122	93.8
	No		8	6.2
Surgery (curative)	Yes		54	41.5
	No		76	58.5
Metastasis^b^	Yes		80	61.5
	No		50	38.5

BMI, body mass index; SD, standard deviation.

a “Religious beliefs” refers to participants' self-reported presence or absence of any religious belief or affiliation, regardless of specific religion or level of religiosity.

b “metastasis” includes both distant metastases and regional lymph node involvement.

### Composition of symptom clusters in patients with GI cancer during radiotherapy

In our previous exploratory study, symptom cluster extraction was performed using exploratory factor analysis. Using exploratory factor analysis, four symptom clusters were identified at each time point during radiotherapy, including the emotion-energy deficiency, eating difficulty, nervous, and numbness-nausea symptom clusters. The composition of each symptom cluster is shown in Table 2.

**Table 2:** Composition of symptom clusters during radiotherapy in patients (*N* ​= ​130).

Symptom cluster	T0			T1			T2		
	Symptom	Rate of occurrence [*n* (%)]	Severity M (P25, P75)	Symptom	Rate of occurrence [*n* (%)]	Severity M (P25, P75)	Symptom	Rate of occurrence [*n* (%)]	Severity M (P25, P75)
**Emotional-energy deficiency symptom cluster**	Lack of appetite	118 (90.8)	3.0 (2.0, 4.0)	Lack of appetite	128 (98.5)	4.0 (3.0, 5.0)	Lack of appetite	126 (96.9)	3.0 (3.0, 4.0)
	Fatigue	128 (98.5)	3.0 (2.0, 4.0)	Fatigue	127 (97.7)	4.0 (4.0, 5.0)	Fatigue	127 (97.7)	3.0 (3.0, 4.0)
	Sadness	129 (99.2)	3.0 (3.0, 4.0)	Sadness	128 (98.5)	4.0 (3.0, 4.0)	Sadness	129 (99.2)	3.0 (3.0, 4.0)
	Distress	129 (99.2)	4.0 (3.0, 4.0)	Distress	129 (99.2)	4.0 (4.0, 5.0)	Distress	129 (99.2)	4.0 (3.0, 4.0)
	Disturbed sleep	121 (93.1)	2.0 (1.0, 3.0)	Disturbed sleep	126 (96.9)	3.0 (3.0, 5.0)	Disturbed sleep	121 (93.1)	2.0 (2.0, 3.0)
	Taste change	77 (59.2)	1.0 (0.0, 2.0)	Taste change	100 (76.9)	2.0 (1.0, 2.0)	–	–	–
	Numbness	48 (37.0)	0.0 (0.0, 2.0)	–	–	–	–	–	–
**Difficulty eating symptom cluster**	Difficulty swallowing	64 (49.2)	0.0 (0.0, 2.0)	Difficulty swallowing	78 (60.0)	2.0 (0.0, 3.0)	Difficulty swallowing	72 (55.4)	1.0 (0.0, 2.0)
	Pain	48 (36.9)	0.0 (0.0, 1.0)	Pain	91 (70.0)	2.0 (0.0, 2.0)	Pain	48 (36.9)	0.0 (0.0, 1.0)
	Nausea	30 (23.1)	0.0 (0.0, 0.0)	–	–	–	Taste change	91 (70.0)	1.0 (0.0, 2.0)
**Nervous system symptom cluster**	Difficulty remembering	116 (89.2)	2.0 (1.0, 4.0)	Difficulty remembering	118 (90.8)	2.0 (1.0, 4.0)	Difficulty remembering	117 (90.0)	2.0 (1.0, 4.0)
	Drowsy	118 (90.8)	1.0 (1.0, 2.0)	Drowsy	125 (96.2)	2.0 (1.0, 3.0)	–	–	–
	–	–	–	Dry mouth	120 (92.3)	3.0 (2.0, 4.0)	Dry mouth	101 (77.7)	1.0 (1.0, 2.0)
**Numbness-nausea symptom cluster**	–	–	–	Numbness	56 (43.1)	0.0 (0.0, 2.0)	–	–	–
	–	–	–	Nausea	91 (70.0)	2.0 (0.0, 2.0)	–	–	–

M, median. “–”means that the symptom is not present at that location.

### Symptom network analysis of patients with GI cancer during radiotherapy

**Fig. 2:**
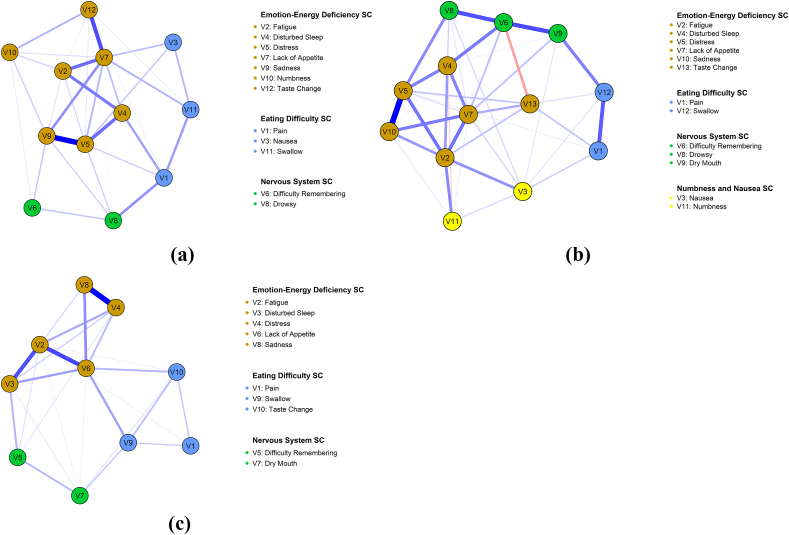
Symptom network of patients during T0-T2. (a) T0; (b) T1; (c) T2 (Panels a, b, and c correspond to the subfigures).

The symptom network diagram provided a visual representation of relationships among symptoms experienced by patients with GI cancer. As shown in Fig. 2, most symptom pairs were positively correlated.

### Analysis of centrality indicators

**Fig. 3:**
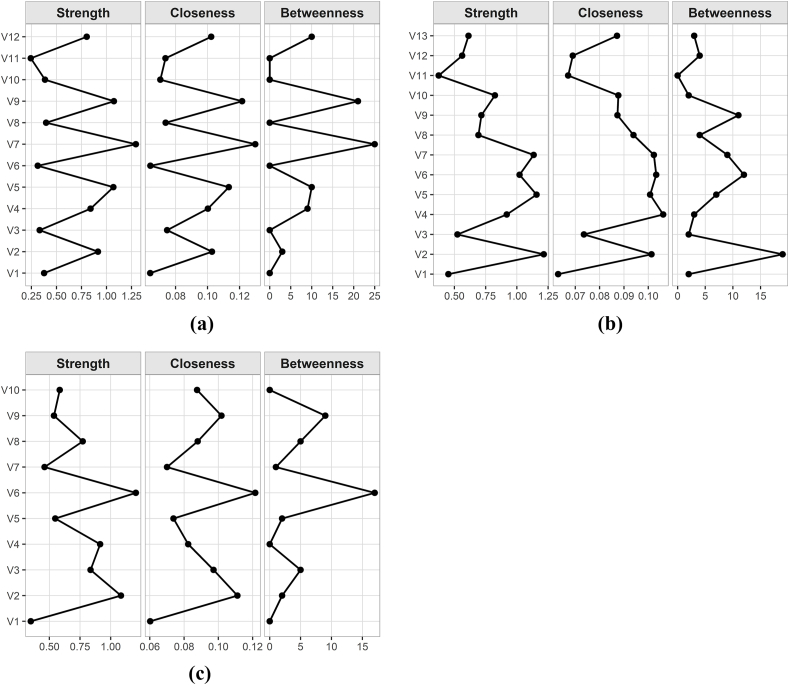
Centrality indicators of symptom network of patients during T0-T2. (a) T0; (b) T1; (c) T2 (Panels a, b, and c correspond to the subfigures). CI, confidence interval.

As shown in Fig. 3, lack of appetite exhibited the highest strength (*r*_S_ ​= ​1.293), closeness (*r*_C_ ​= ​0.012) and betweenness (*r*_B_ ​= ​50) at T0 were all the highest. At T1, fatigue showed the highest strength (*r*_S_ ​= ​1.215) and betweenness (*r*_B_ ​= ​38), whereas sleep disturbance demonstrated the highest closeness (*r*_C_ ​= ​0.009). At T2, lack of appetite again exhibited the highest strength (*r*_S_ ​= ​1.180), closeness (*r*_C_ ​= ​0.013), and betweenness (*r*_B_ ​= ​36).

### Stability and accuracy analysis of symptom network structures

**Fig. 4:**
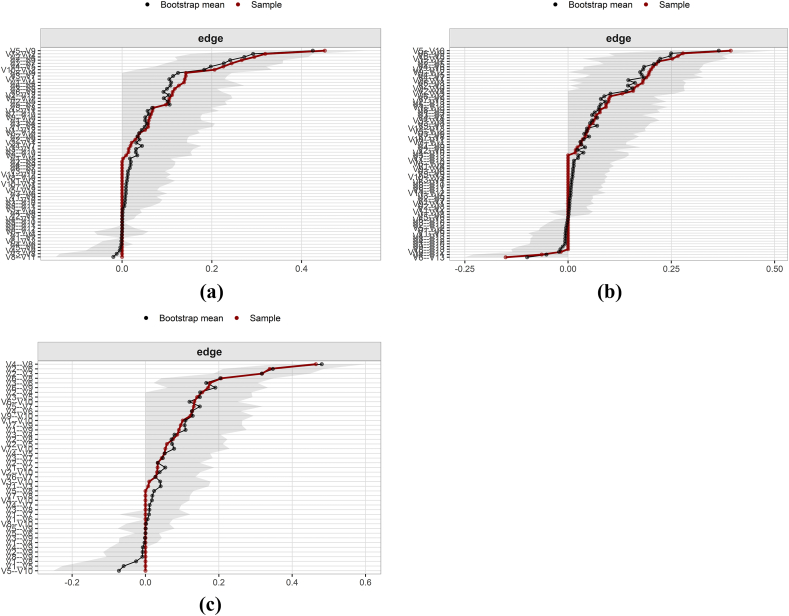
Bootstrap analysis results of patients edge weights during T0-T2. (a) T0; (b) T1; (c) T2 (Panels a, b, and c correspond to the subfigures). CI, confidence interval. The red line indicates the edge weight value, and the gray area indicates the 95% CI; the smaller the gray area interval and the smaller the 95% CI, the higher the accuracy of the edge weights.

Fig. 4 showed that some edge weights had large CI ranges but small overall gray area bands, indicating high accuracy of edge weight estimates across all time points.

**Fig. 5:**
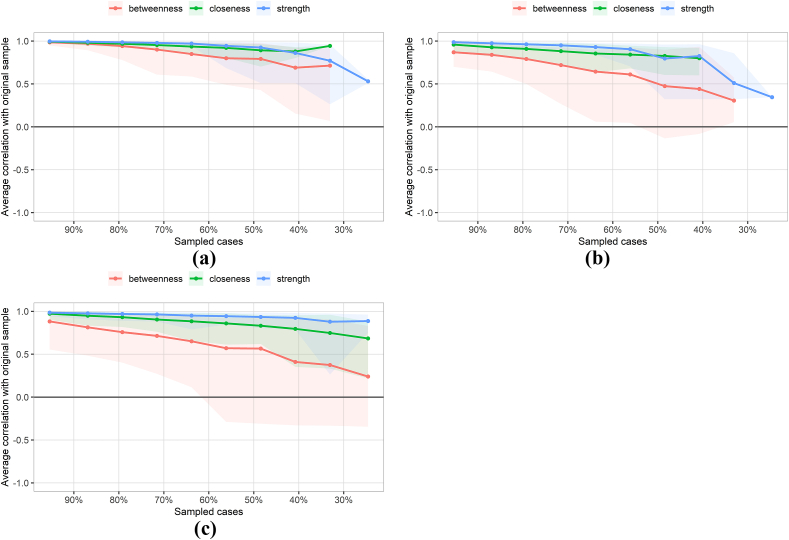
Stability analysis of symptom network centrality indicators during T0-T2. (a) T0; (b) T1; (c) T2 (Panels a, b, and c correspond to the subfigures). CI, confidence interval. The red area indicates the stability of betweenness, the green area indicates the stability of closeness, and the blue area indicates the stability of strength; the smaller the area, the smaller the 95% CI, and the higher the stability of the centrality indicator.

Fig. 5 showed that strength exhibited relatively higher stability across time points, even as sample size decreased. At T0, the CS-coefficients for betweenness, closeness, and strength were 0.285, 0.131 and 0.362, respectively. Stability was considered acceptable for betweenness and strength, but not for closeness. At T1, the CS-coefficients for betweenness, closeness and strength were 0.046, 0.208 and 0.362, respectively. Only strength demonstrated acceptable stability. At T2, the CS-coefficients were 0.046 for both betweenness and closeness, and 0.515 for strength. Again, only strength showed acceptable stability. Therefore, strength at each time point and betweenness at T0 were selected as the primary metrics for identifying core symptom clusters in patients with GI cancer.^33^

### Difference test of edge weight and node strength

**Fig. 6:**
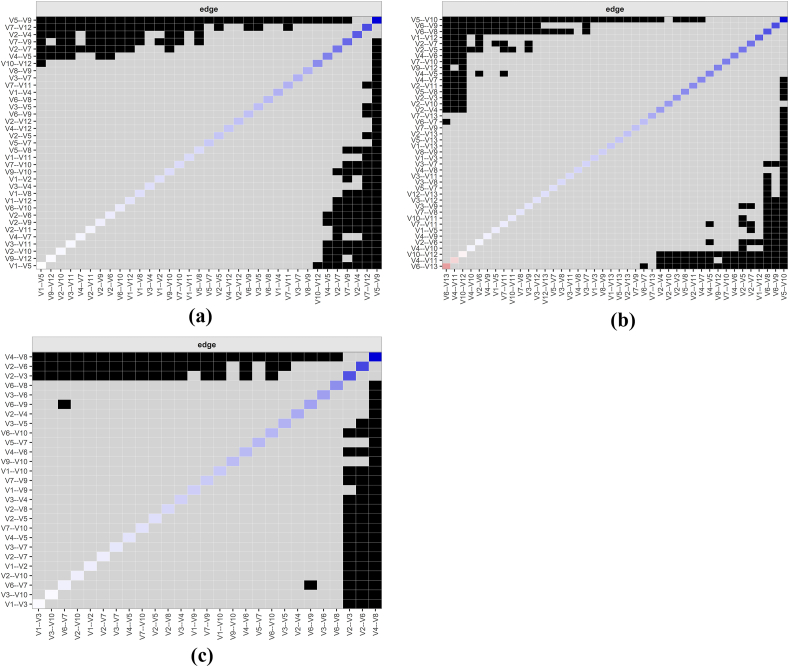
Bootstrap difference test for edge weights during T0-T2. (a) T0; (b) T1; (c) T2 (Panels a, b, and c correspond to the subfigures). Black areas represent statistically significant differences in the weights at the edges of the two connecting lines, and gray areas represent statistically insignificant differences.

The bootstrap difference test for edge weights in Fig. 6 showed that at T0, the edges between V5 (distress) and V9 (sadness), V7 (lack of appetite) and V12 (taste change), V2 (fatigue) and V4 (disturbed sleep), and V7 (lack of appetite) and V9 (sadness) showed statistically significant differences from other edges in the network. At T1, significant edge weight differences were observed for V5 (distress)–V10 (sadness), V6 (difficulty remembering)–V9 (dry mouth), and V6 (difficulty remembering)–V8 (drowsy). At T2, the edges between V4 (distress) and V8 (sadness), V2 (fatigue) and V6 (lack of appetite), and V2 (fatigue) and V3 (disturbed sleep) also showed significant differences from other edge weights.

**Fig. 7:**
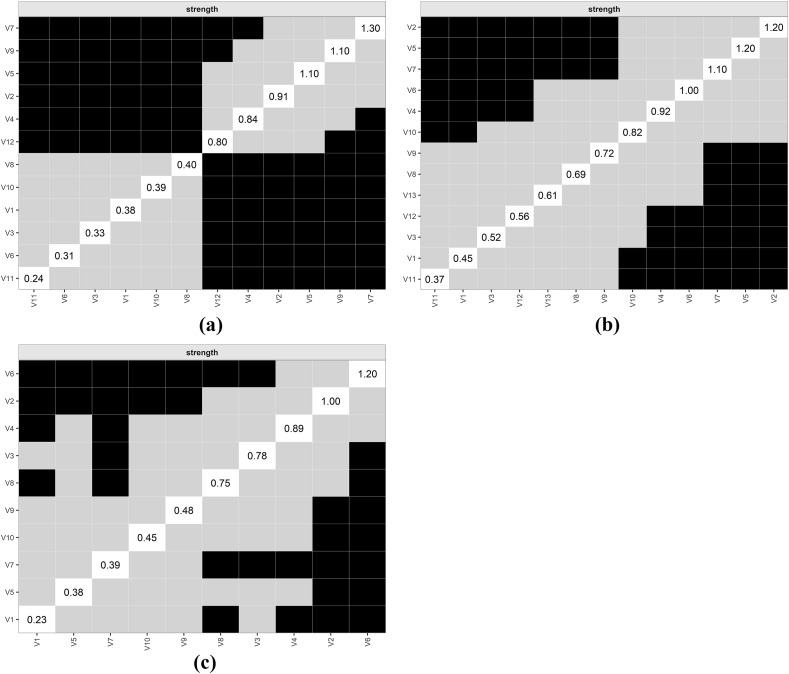
Bootstrap difference test for node strength during T0-T2. (a) T0; (b) T1; (c) T2 (Panels a, b, and c correspond to the subfigures). The black area represents statistically significant differences in the strength of the two nodes, and the gray area represents statistically insignificant differences.

The bootstrap difference test of node strength in Fig. 7 showed that at T0, approximately half of the nodes did not significantly differ in strength. However, V7 (lack of appetite), which had the highest strength, was significantly stronger than other nodes (DTs ​= ​1.30). At T1, some nodes did not significantly differ in strength, while V2 (fatigue) and V5 (distress) demonstrated significantly greater strength than other nodes (DTs ​= ​1.20). At T2, V6 (lack of appetite) exhibited significantly greater node strength than the other nodes (DTs ​= ​1.20).

### Screening of core symptom clusters during radiotherapy in patients with GI cancers

According to findings from our research team’ prior exploratory factor analysis, distress, sadness, fatigue, and loss of appetite were among the most frequently occurring and severe symptoms at each radiotherapy time point in patients with gastrointestinal cancer, consistently appearing within the top five rankings. In this study, centrality analysis revealed that lack of appetite had the highest strength at both T0 and T2, identifying it as the core symptom at these time points. At T1, fatigue showed the highest strength and was considered the core symptom. Additionally, lack of appetite had the highest betweenness at T0, indicating its role as a bridge symptom during that period.

In summary, among the three symptom clusters consistently present across all time points, the emotion–energy deficiency cluster not only included symptoms with the highest incidence and severity but also those with the highest centrality indicators. This cluster was preliminarily identified as the core symptom cluster during radiotherapy. As this cluster consistently included lack of appetite, fatigue, sadness, distress, and disturbed sleep, the emotion-energy deficiency symptom cluster comprising these symptoms was ultimately identified as the core symptom cluster during radiotherapy.

### Longitudinal changes in quality of life during radiotherapy in patients with GI cancer

Longitudinal changes in quality of life during radiotherapy for patients with GI cancer are presented in Table 3. Except for shortness of breath and constipation, differences in quality-of-life scores across time points were statistically significant in patients receiving radiotherapy for GI cancer (*P* ​< ​0.05). Role functioning and overall quality of life declined from T0, reached their lowest point at T1, and improved by T2 (*P* ​< ​0.05). Fatigue and lack of appetite increased from T0, peaked at T1, and decreased by T2 (*P* ​< ​0.05).

**Table 3:** Longitudinal comparison of scores on dimensions of quality of life (*N* ​= ​130).

Dimension	Category	T0	T1	T2	Wald *χ*^2^	*P*	Post-hoc test
		M (P25, P75)	M (P25, P75)	M (P25, P75)			
**Functional dimension**	Physical function	93.3 (93.3, 100.0)	93.3 (86.7, 93.3)	93.3 (91.7, 93.3)	25.189	< 0.001	T0 > T1, T1 ​< ​T2
	Role function	66.7 (66.7, 83.3)	66.7 (66.7, 66.7)	66.7 (66.7, 83.3)	104.246	< 0.001	T0 > T2 ​> ​T1
	Emotional function	66.7 (50.0, 75.0)	62.5 (50.0, 66.7)	75.0 (58.3, 75.0)	66.471	< 0.001	T0 > T1, T1 ​< ​T2
	Cognitive function	83.3 (66.7, 83.3)	66.7 (66.7, 83.3)	66.7 (66.7, 83.3)	11.454	0.003	T0 ​> ​T1
	Social function	66.7 (66.7, 66.7)	66.7 (50.0, 66.7)	66.7 (66.7, 66.7)	70.853	< 0.001	T0 > T1, T1 ​< ​T2
**Symptom dimension**	Fatigue	33.3 (11.1, 33.3)	33.3 (33.3, 33.3)	33.3 (22.2, 33.3)	95.392	< 0.001	T0 ​< ​T2 ​< ​T1
	Nausea/Vomiting	0.0 (0.0, 0.0)	0.0 (0.0, 16.7)	0.0 (0.0, 0.0)	8.317	0.016	T1 ​> ​T2
	Pain	0.0 (0.0, 16.7)	16.7 (0.0, 16.7)	0.0 (0.0, 16.7)	87.142	< 0.001	T0 < T1, T1 > T2
**Single symptoms**	Shortness of breath	0.0 (0.0, 0.0)	0.0 (0.0, 0.0)	0.0 (0.0, 0.0)	0.789	0.674	–
	Insomnia	33.3 (0.0, 33.3)	33.3 (33.3, 66.7)	33.3 (33.3, 33.3)	69.689	< 0.001	T0 < T1, T1 > T2
	Lack of appetite	33.3 (33.3, 33.3)	33.3 (33.3, 66.7)	33.3 (33.3, 33.3)	58.281	< 0.001	T0 ​< ​T2 ​< ​T1
	Constipation	0.0 (0.0, 0.0)	0.0 (0.0, 0.0)	0.0 (0.0, 0.0)	1.153	0.562	–
	Diarrhea	0.0 (0.0, 0.0)	0.0 (0.0, 0.0)	0.0 (0.0, 0.0)	7.775	0.021	T1 ​> ​T2
	Financial strain	33.3 (33.3, 33.3)	33.3 (33.3, 33.3)	33.3 (33.3, 33.3)	13.220	0.001	T0 ​< ​T1, T0 ​< ​T2
**Overall quality of life dimension**	–	50.0 (50.0, 66.7)	50.0 (33.3, 50.0)	50.0 (50.0, 58.3)	298.764	< 0.001	T0 > T2 ​> ​T1

M, median.

### Correlation between core symptom clusters and quality of life at various time points during radiotherapy in patients with GI cancer

Table 4 showed that the severity scores of the core symptom cluster during radiotherapy were negatively correlated with functional dimension scores and global quality of life scores from T0 to T2 (*r* ​= ​−0.331 to −0.700, *P* ​< ​0.01).

**Table 4:** Correlation between core symptom clusters and quality of life (*N* ​= ​130, *r*).

Dimension	Category	Phase	Core symptom cluster		
			T0	T1	T2
**Functional dimension**	Physical function	T0	−0.513^a^		
		T1		−0.331^a^	
		T2			−0.440^a^
	Role function	T0	−0.585^a^		
		T1		−0.573^a^	
		T2			−0.641^a^
	Emotional function	T0	−0.541^a^		
		T1		−0.626^a^	
		T2			−0.700^a^
	Cognitive function	T0	−0.391^a^		
		T1		−0.478^a^	
		T2			−0.407^a^
	Social function	T0	−0.432^a^		
		T1		−0.591^a^	
		T2			−0.653^a^
**Overall quality of life dimension**		T0	−0.444^a^		
		T1		−0.434^a^	
		T2			−0.532^a^

a *P* ​< ​0.01.

## Discussion

This study is novel in applying longitudinal symptom network analysis specifically to patients with GI cancers undergoing radiotherapy. In contrast to prior studies that focused on single time-point assessments or chemotherapy cohorts, this study investigates the dynamic interrelationships among symptoms within the context of radiotherapy. By examining symptom networks across treatment stages and linking them to patients’ quality of life, this study provides a conceptual foundation for developing stage-specific, precision-guided symptom management strategies in clinical practice.

### Lack of appetite and fatigue are core symptoms

The results of this study showed that lack of appetite had the highest strength at T0 and T2, while fatigue had the highest strength at T1, identifying them as core symptoms at their respective time points. These findings are consistent with Li et al.^34^ and Rha et al.^35^ Core symptoms have the strongest association with other symptoms in the symptom network. Targeted alleviation of core symptoms may reduce the severity of related symptoms and enhance intervention effectiveness.^35^^,^^36^ Therefore, interventions targeting lack of appetite and fatigue should be prioritized during radiotherapy.

Decreased appetite has been shown to exacerbate protein and energy consumption, leading to insufficient nutrient reserves, triggering micronutrient deficiencies, muscle weakness, decreased quality of life, and increased mortality.^37^ In addition, appetite has a significant impact on the quality of life of patients through physiological and psychological mechanisms. Thus, appetite should be considered as a core indicator of quality of life in patients with GI cancer. Health care professionals should systematically assess the potential causes of decreased appetite and effectively improve nutritional status by optimizing the dietary structure, formulating a personalized treatment plan, implementing psychological interventions and appetite stimulation and other comprehensive measures.^38^

Fatigue is one of the most frequently reported symptoms during cancer treatment.^39^ Studies indicate that up to 40% of patients experience fatigue at diagnosis, and the incidence can rise to over 90% during radiotherapy.^40^ In a longitudinal study of chemotherapy patients, fatigue was strongly associated with symptoms such as pain and anxiety, and was identified as a core indicator in symptom assessment systems.^35^ Cancer-related fatigue is a persistent and distressing subjective experience of physical, emotional, and/or cognitive tiredness that is disproportionate to activity and unrelieved by rest.^41^ Although often ignored by both patients and health care professionals, fatigue poses direct threats to the health of patients, including reduced quality of life, delayed recovery, and an increased risk psychological problems such as depression.^42^ Therefore, health care professionals should closely monitor fatigue in patients with GI cancer, help patients to adjust their mindset, avoid overwork, encourage appropriate activities, regularly assess the fatigue level of patients, and encourage proactive symptom reporting, so as to realize the normalized management of fatigue.

In addition, lack of appetite also functioned as a bridge symptom at T0, acting as a key connector among symptom clusters, and its intervention may disrupt symptom associations.^43^^,^^44^ Therefore, it is recommended that lack of appetite be targeted in pre-radiotherapy symptom management to weaken the transmission of bridge symptoms and improve the efficiency of symptom management.

### Emotion-energy deficiency symptom cluster is the core symptom cluster of patients with GI cancer during radiotherapy

The results of this study showed that lack of appetite, fatigue, sadness, distress, and disturbed sleep within the emotion-energy deficiency symptom cluster remained stable and persistent throughout all time points during radiotherapy, and encompassed symptoms with the highest incidence and severity (distress), highest strength (lack of appetite and fatigue), and highest betweenness (lack of appetite at T0). Accordingly, the emotion-energy deficiency symptom cluster was identified as the core symptom cluster in this study, which was similar to the core symptom cluster (pain, fatigue, disturbed sleep, distress and sadness) identified by Guo et al.^45^ in patients with hematological tumors undergoing chemotherapy. In a cross-sectional study involving 202 patients with GI cancer, Wang et al.^11^ identified the core symptom cluster as a psychoemotional cluster comprising numbness, distress and sadness. Although this finding aligns with the current study, some differences were noted. These discrepancies may be attributable to differences in treatment modalities among study populations. Previous studies^11^^,^^46^ have shown strong associations among fatigue, sleep disturbance, distress and sadness, the targeted management of which can significantly reduce the symptom burden and improve patients’ quality of life.

### Longitudinal changes in quality of life during radiotherapy in patients with GI cancer

The results of this study showed that differences in all quality-of-life dimensions, except for shortness of breath and constipation, were statistically significant across different radiotherapy time points (*P* ​< ​0.05), consistent with the findings of Wang et al.^47^ Scores in functional dimensions and overall quality of life, except for cognitive function, exhibited a downward trend followed by improvement. This may be attributed to the cumulative adverse effects of prolonged radiotherapy, which may lead to fatigue, appetite loss, and sleep disturbance, subsequently impairing somatic function.^48^^,^^49^ Simultaneously, severe adverse effects, insufficient disease-related knowledge, and uncertainty about prognosis can contribute to distress, sadness, and anxiety,^50^ thereby affecting emotional function. In addition, the radiotherapy process usually lasts 1–2 months, during which patients may experience emotional and social isolation due to prolonged treatment stress and limited social interactions.^51^ This may weaken family and social roles and impair role and social functioning, ultimately reducing overall quality of life. As radiotherapy concluded, adverse effects gradually diminished and patients returned to normal life, with significant improvement in their functions and quality of life. Among the symptom dimensions and single symptom entries, the scores of fatigue and loss of appetite increased during treatment and declined post-radiotherapy. This may be partly attributed to cumulative symptom burden during radiotherapy, potentially influenced by prior treatments, comorbidities, and age-related vulnerability. These factors may have contributed to treatment discontinuation and declining quality of life in some patients. Following treatment, these adverse reactions diminished, and the severity of symptoms decreased or resolved. This suggests that health care professionals should pay close attention to the occurrence of adverse reactions during radiotherapy and take appropriate measures to deal with them in a timely manner, address multidimensional care needs, proactive education regarding disease-related information, monitor emotional well-being, and promote overall quality of life.

### Correlation between core symptom clusters and quality of life during radiotherapy for patients with GI cancer

The results of this study showed that core symptom cluster severity scores were negatively correlated with all quality-of-life dimensions, except for symptom dimensions and single symptom items(*r* ​= ​−0.331 to −0.700, *P* ​< ​0.01). This suggests that higher severity of core symptom clusters is associated with poorer quality of life, consistent with previous studies.^6^^,^^52^ This indicates that the core symptom cluster during radiotherapy in patients with GI cancer is an important factor affecting patients' quality of life, and that effective, comprehensive, and systematic management strategies are essential for improving patient outcomes. The strongest negative correlations were observed between core symptom cluster severity and role functioning (T0), as well as emotional functioning (T1 and T2), which aligns with the findings of Lin et al.,^46^ indicating that core symptom clusters had the greatest impact on patients' role and emotional functioning during radiotherapy. Therefore, in addition to managing core symptom clusters, clinical staff should pay close attention to changes in patients’ roles and emotional states, encourage emotional expression, and deliver targeted supportive care based on individual psychological experiences to improve overall quality of life.

### Implications for nursing practice and research

The study found that loss of appetite and fatigue were core symptoms, and that the emotion-energy deficiency symptom cluster was the core symptom cluster, which was negatively associated with quality of life. These findings may assist clinical nurses in understanding the complex interrelationships among symptoms in patients with GI cancer undergoing radiotherapy. Future interventions could target this symptom cluster and evaluate their effectiveness in reducing symptom burden and improving patients’ quality of life.

### Limitations

This study has several important limitations. First, the study was conducted at a single tertiary hospital in Shandong Province using a convenience sampling method, which may have introduced selection bias and limited the generalizability of the findings. Patients from a single center may share similar clinical characteristics and treatment contexts, potentially influencing symptom perception and self-reporting. To mitigate this limitation, future studies should adopt multicenter designs and implement stratified sampling strategies to enhance external validity. Second, the sample was predominantly composed of patients with esophageal cancer, thereby limiting the applicability of the findings to the broader gastrointestinal cancer population. Future research should recruit more different participants across various GI cancer types to ensure wider representativeness. In addition, the absence of an independent validation cohort reduced the reproducibility and robustness of the symptom network model. To strengthen the reliability of network structures, subsequent studies should incorporate independent validation cohorts. Finally, the inclusion of patients with heterogeneous gastrointestinal cancer types and stages may have introduced sample variability; however, no subgroup or covariate analyses were conducted to evaluate the influence of clinical characteristics on symptom trajectories. Future research should therefore employ stratified or covariate-adjusted analyses to clarify how clinical characteristics shape symptom dynamics and affect patient outcomes.

## Conclusions

Multiple symptom clusters were identified in patients with gastrointestinal cancer during radiotherapy, among which the emotion-energy deficiency cluster served as the core symptom cluste and was negatively associated with quality of life. Accurate identification of core symptom clusters during radiotherapy is critical for the development of targeted symptom interventions. Health care professionals should adopt a multifaceted approach to develop tailored management strategies for the core symptom cluster and implement personalized interventions to reduce symptom burden and improve patient's quality of life.

## CRediT authorship contribution statement

**Mengjia Liu:** Conceptualization, Methodology, Software, Formal analysis, Writing – Original Draft. **Huiwen Ma**: Writing – Original Draft, Writing – Review & Editing, Visualization. **Yaxin Chang**: Investigation, Formal Analysis. **Hong Li**: Validation, Investigation. **Ruiqi Chen**: Investigation, Data Curation. **Weizheng Cui:** Validation, Data Curation. **Zhaoxia Yang:** Visualization, Supervision. **Ke Wang:** Conceptualization, Methodology, Supervision. All authors have read and approved the final manuscript.

## Ethics statement

The study was approved by the Ethics Committee of the Second Affiliated Hospital of Shandong First Medical University (Approval No. 2023-H-079) and was conducted in accordance with the 1964 Helsinki Declaration and its later amendments or comparable ethical standards. All participants provided written informed consent.

## Data availability statement

The data that support the findings of this study are available from the corresponding author, Z.Y., upon reasonable request.

## Declaration of generative AI and AI-assisted technologies in the writing process

During the preparation of this work the authors used ChatGPT in order to improve language and readability. After using this tool, the authors reviewed and edited the content as needed and take full responsibility for the content of the publication.

## Funding

This study was supported by the Tai'an City Agricultural and Social Development Science and Technology Innovation Project (Grant No. 2024NS201). The funders had no role in considering the study design or in the collection, analysis, interpretation of data, writing of the report, or decision to submit the article for publication.

## Declaration of competing interest

The authors declare no conflict of interest.
